# Association of renal function with muscle strength in Korean adults: A population-based study using the Korea National Health and Nutrition Examination Surveys (KNHANES) from 2014 to 2018

**DOI:** 10.1097/MD.0000000000031014

**Published:** 2022-10-14

**Authors:** Young-Mo Yang, Eun Joo Choi

**Affiliations:** a Department of Pharmacy, College of Pharmacy, Chosun University, Gwangju, South Korea.

**Keywords:** age, body mass index, gender, hand grip strength, Koreans, renal function

## Abstract

Hand grip strength (HGS), a simple measure of upper limb muscle function, can be used to assess overall muscular strength, and reduced HGS in patients with poor renal functions has been observed. This study examined the associations between renal function and HGS, a surrogate marker of muscular strength, among a stratified sample of Korean adults. This study obtained data from the Korea National Health and Nutrition Examination Survey from 2014 to 2018, a cross-sectional and nationally representative survey conducted by the Korea Centers for Diseases Control and Prevention. In men, low muscle strength (LMS) and normal muscle strength (NMS) were defined as HGS < 28.9 kg and HGS ≥ 28.9 kg, respectively. In women, LMS and NMS were considered as HGS < 16.8 kg and HGS ≥ 16.8 kg, respectively. Of the 25,746 subjects in this study, there were 3603 (14.0%) and 22,143 (86.0%) subjects who displayed LMS and NMS, respectively. Subjects with estimated glomerular filtration rate (eGFR) < 60 mL/min/1.73 m^2^ had a higher risk of developing LMS than those with eGFR ≥ 60 mL/min/1.73 m^2^ after adjusting for age (odds ratio, 1.772; 95% CI, 1.498–2.096); the significant differences remained after adjusting for other factors including age. Similar tendencies were shown in men and women when analyzed according to gender; however, the risk of developing LMS was higher in men than in women. Results showed that decreased renal function was likely to contribute to an increased prevalence of LMS based on HGS. This association may assist in developing better strategies to estimate renal function in clinical or public health practice.

## 1. Introduction

Hand grip strength (HGS), which is a simple measure of upper limb muscle function, can be used as an indicator of overall muscular strength. Further, several studies have shown that it is negatively linked with all-cause mortality, functional and cognitive impairments, physical disabilities, and nutritional status.^[1–3]^ Reduced HGS in patients with poor renal functions was observed in previous studies.^[4–7]^ According to the study by Hiraki and colleagues, HGS was significantly lower in patients with chronic kidney disease (CKD) stage 4 or 5 than in those with CKD stage 2 or 3.^[6]^ Amparo and colleagues reported that lower HGS in patients with CKD stages 2–5 was associated with reduced kidney function, lower serum albumin, and worse evaluation by the Malnutrition-Inflammation Score that is used as a nutritional assessment tool.^[8]^

CKD may be caused by various risk factors such as race, gender, age, low birth weight, family history, smoking, obesity, nephrotoxic medications, hypertension (HTN), and diabetes mellitus (DM).^[9,10]^ In particular, progressive reductions in the glomerular filtration rate and renal blood flow are shown to be associated with aging, which may lead to age-associated loss of renal function.^[11,12]^ Decreased renal function can also occur in those who are overweight or obese, irrespective of the presence of HTN, DM, or cardiovascular disease.^[13]^ Additionally, gender differences in the progression of CKD to end-stage renal disease and hemodialysis may exist.^[14]^

Consequently, age, obesity, and gender as risk factors for the development of CKD may play an important role in determining the prevalence rate of low muscle strength (LMS) in patients with reduced renal functions. However, to our knowledge, limited studies have investigated the relationships between renal function and muscle strength in Korean adults. Therefore, this study aimed to examine the associations between renal function and HGS, a surrogate marker of muscular strength, among Korean adults stratified by age, body mass index (BMI), and gender.

## 2. Methods

### 2.1. Study population

**Figure 1:**
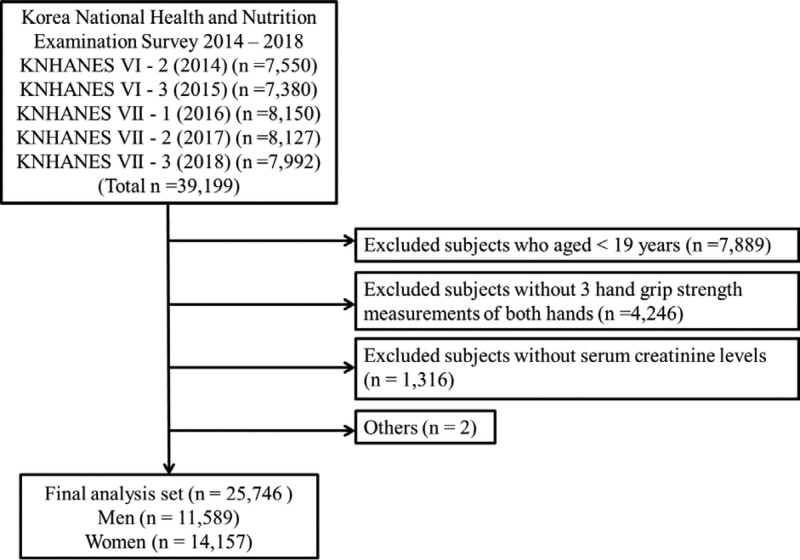
. Flow chart of selecting study subjects from the Korea National Health and Nutrition Examination Survey (KNHANES) 2014–2018.

This study was conducted using the data obtained from the Korea National Health and Nutrition Examination Survey (KNHANES) data from 2014 to 2018, a cross-sectional and nationally representative survey performed by the Korea Centers for Diseases Control and Prevention. The KNHANES data consisted of a health interview, health examination, and nutrition survey. The data were acquired by conducting household interviews, and the standardized physical examinations were implemented at mobile examination centers. The procedures for conducting the KNHANES were approved by the Korea centers for diseases control and prevention Institutional Review Board, and informed written consent was acquired from all survey participants. First, among all participants, the subjects aged < 19 years were excluded. Second, those without 3 grip strength measurements of both hands were excluded. Third, those without serum creatinine levels were excluded. Ultimately, among 39,199 subjects, 25,746 adults (11,589 men and 14,157 women) were included in the present study (Fig. 1). Ethical approval for the present study was obtained by the institutional review board of Chosun University (2-1041055-AB-N-01-2021-74).

### 2.2. HGS measurements

HGS was measured with a digital grip strength dynamometer (TKK 5401 Grip-D; Takei, Niigata, Japan) that has an adjustable grip span. This could measure HGS between 5.0 and 100.0 kg, and the minimum measurement unit was 0.1 kg. During the measurement, participants stood upright with their head up, and their arms remained in a neutral and comfortable position with their elbows fully extended. They held the dynamometer in the testing hand with 90 degrees of flexion at the index finger. They conducted 3 trials for each hand using the dominant hand first, and they continuously squeezed the grip for at least 3 seconds. While squeezing the grip, they did not swing the dynamometer and hold their breath. They rested for approximately 60 seconds between the trials. The average data of 3 trials for the dominant hand was used in the statistical analysis. Based on the previous study conducted with the KNHANES data from 2014 to 2015, participants were divided into 2 groups: LMS and normal muscle strength (NMS). In men, LMS was defined as HGS < 28.9 kg, and NMS was defined as HGS ≥ 28.9 kg. In women, LMS was considered as HGS < 16.8 kg, and NMS was considered as HGS ≥ 16.8 kg.^[15]^

### 2.3. Anthropometric and laboratory data

Body weight and height were measured to the nearest 0.1 kg and 0.1 cm, respectively, while participants wore light indoor clothing without shoes. BMI was calculated by dividing weight in kilograms by height in meters squared (kg/m^2^). BMI < 18.5 kg/m^2^ was defined as underweight, BMI between ≥ 18.5 kg/m^2^ and < 25.0 kg/m^2^ as normal weight, and BMI ≥ 25.0 kg/m^2^ as overweight or obese.^[16]^

Blood samples were collected after at least an 8-hour fast, and random spot urine samples were obtained from participants. The samples were properly processed, immediately refrigerated, and transported in cold storage to the central laboratory within 24 hours. Serum creatinine and blood urea nitrogen were measured using the Hitachi Automatic Analyzer 7600-210 (Hitachi High-Tech Corp., Tokyo, Japan). Urine dipstick analysis was conducted using the Urisys 2400 automated urine analyzer (Roche Diagnostics GmbH, Mannheim, Germany), and proteinuria was considered as trace or greater.

### 2.4. Renal function measurements

An estimated glomerular filtration rate (eGFR) was calculated with the CKD Epidemiology Collaboration equation (Table 1).^[17]^ Participants were grouped based on their eGFR levels as follows: stage 1 = eGFR ≥ 90 mL/min/1.73 m^2^, stage 2 = eGFR 60–89 mL/min/1.73 m^2^, stage 3a = eGFR 45–59 mL/min/1.73 m^2^, stage 3b = eGFR 30–44 mL/min/1.73 m^2^, and stage 4/5 = eGFR < 30 mL/min/1.73 m^2^.^[18]^ Additionally, they were re-grouped according to their renal function stages (i.e., stages 1–2 and stages 3a–5).

**Table 1:** The CKD-EPI equation for estimating GFR.

Race & sex	Scrlevel (mg/dL)	Equation
Black		
Women	≤0.7	GFR = 166 * (Scr/0.7^)-0.329^ * (0.993)^Age^
	>0.7	GFR = 166 * (Scr/0.7^)-1.209^ * (0.993)^Age^
Men	≤0.9	GFR = 163 * (Scr/0.9^)-0.411^ * (0.993)^Age^
	>0.9	GFR = 163 * (Scr/0.9^)-1.209^ * (0.993)^Age^
White or others		
Women	≤0.7	GFR = 144 * (Scr/0.7^)-0.329^ * (0.993)^Age^
	>0.7	GFR = 144 * (Scr/0.7^)-1.209^ * (0.993)^Age^
Men	≤0.9	GFR = 141 * (Scr/0.7^)-0.411^ * (0.993)^Age^
	>0.9	GFR = 141 * (Scr/0.7^)-1.209^ * (0.993)^Age^

CKD-EPI, chronic kidney disease epidemiology collaboration; GFR, glomerular filtration rate; Scr, serum creatinine.

### 2.5. HTN and DM

Blood pressure measurements, using a standard mercury sphygmomanometer, were performed on the right arm of the participants after they were seated for at least 5 minutes. Three measurements were recorded for all participants at 5-minute intervals, and the mean of the second and third measurements was used in the analysis. HTN was defined as systolic blood pressure ≥ 140 mm Hg, diastolic blood pressure ≥ 90 mm Hg, or use of antihypertensive medications independently of blood pressure.^[19]^

After at least an 8-hour fasting period, blood glucose was measured using the Hitachi Automatic Analyzer 7600-210 (Hitachi High-Tech Corp., Tokyo, Japan). DM was defined as fasting glucose ≥ 126 mg/dL.^[20]^ The participants with a known diagnosis of DM treated with hypoglycemic agents and/or insulin, regardless of the fasting glucose level, were also included in the DM group.

### 2.6. Other variables

The subjects answered a self-reported questionnaire on age, socioeconomic variables (i.e., household income and educational level), and lifestyle variables (i.e., smoking status, alcohol consumption, and physical activity). Household income was categorized into quartile ranges based on the monthly average family equivalent income as follows: low, lower middle, higher middle, and high. Educational level was divided into the following 4 groups: elementary school graduation or lower, middle school graduation, high school graduation, and college graduation or higher. Smoking status was divided into 3 groups. nonsmokers were defined as the participants who had never smoked in their lifetime, and past-smokers were defined as those who had smoked in the past but did not smoke at the time the survey was conducted. Current smokers were defined as those who kept smoking daily or often at the time the survey was conducted. Heavy alcohol drinking was established when women and men had at least 5 and 7 drinks, respectively, at one time, more than twice per week. Physical activity during work, transport, and leisure time was assessed based on the total time spent in physical activity per week and the intensity of the physical activity. To meet recommendations on physical activity for health, participants were expected to complete at least 150 minutes/week of moderate-intensity physical activity, 75 minutes/week of vigorous-intensity physical activity, or an equivalent combination of moderate- and vigorous-intensity physical activity achieving ≥ 600 MET-minutes/week.

### 2.7. Statistical analysis

All statistical analyses were conducted with IBM SPSS version 20.0 statistical software (IBM Corp., Armonk, NY) using the KNHANES sampling weights to calculate the representative estimates of the general Korean population. Data were analyzed using a complex survey design considering stratified variables, cluster variables, and weighted variables. A *P* value <.05 was considered statistically significant. The participants included in the analysis were divided based on gender and muscle strength. The chi-square test was used to present categorical variables as frequency and percentage (%), and an independent *t* test was utilized to report continuous variables as mean and standard deviation. Logistic regression analysis for complex sampling adjusted for selected variables was utilized to assess the effects of renal functions (with participants who had eGFR ≥ mL/min/1.73 m^2^ as the reference group) on muscle strength according to gender, age (<65 years vs ≥65 years), and BMI (<25.0 kg/m^2^ vs ≥25.0 kg/m^2^), and the results were presented as odds ratios (ORs) with 95% confidence intervals (CIs).

## 3. Results

Of 25,746 subjects included in this study, there were 3603 (14.0%) and 22,143 (86.0%) subjects who displayed LMS and NMS, respectively. The characteristics of these participants are presented in Table 2. Mean ages of the subjects with LMS and NMS were 58.6 (0.5) and 44.6 (0.2) years, respectively. Mean dominant HGS levels of the subjects with LMS and NMS were 18.4 (0.1) kg and 32.7 (0.1) kg, respectively. The prevalence rates of hypertension (41.3% vs 24.0%), diabetes (18.5% vs 8.6%), and eGFR < 60 mL/min/1.73 m^2^ (8.6% vs 1.7%) were higher in subjects with LMS than in those with NMS. All of the differences, except for that of serum creatinine levels, were statistically significant. Similar results were shown in the subgroup analyses according to gender (Table 3).

**Table 2:** Characteristics of the study population.

Characteristic	LMS(N = 3603)		NMS(N = 22,143)		*P* value
	Weighted% (SE)	UnweightedN	Weighted% (SE)	UnweightedN	
Age (yrs), mean (SE)	58.6 (0.5)	-	44.6 (0.2)	-	<.0001
< 65	51.3 (1.1)	1382	89.5 (0.2)	18,223	<.0001
≥ 65	48.7 (1.1)	2221	10.5 (0.2)	3920	
Household income					
Low	31.8 (1.0)	1102	23.8 (0.4)	5507	<.0001
Lower middle	24.2 (0.9)	887	25.3 (0.4)	5554	
Higher middle	22.0 (0.8)	792	25.4 (0.4)	5704	
High	21.9 (0.9)	794	25.4 (0.5)	5753	
Educational level					
≤Elementary school	39.7 (1.2)	1607	10.8 (0.3)	3226	<.0001
Middle school	11.2 (0.7)	399	8.2 (0.2)	2074	
High school	27.1 (1.0)	727	37.8 (0.5)	7354	
≥College	22.0 (1.0)	609	43.2 (0.5)	8341	
Weight (kg), mean (SE)	59.7 (0.3)	-	66.0 (0.1)	-	<.0001
Height (cm), mean (SE)	159.0 (0.2)	-	165.7 (0.1)	-	<.0001
BMI (kg/m^2^), mean (SE)	23.6 (0.1)	-	23.9 (0.0)	-	<.0001
<25	68.9 (1.0)	2461	65.4 (0.4)	14,428	.0010
≥25	31.1 (1.0)	1120	34.6 (0.4)	7689	
Smoking					
Never	63.1 (1.1)	2198	55.3 (0.4)	12,879	<.0001
Past	22.2 (0.8)	842	21.3 (0.4)	4575	
Current	14.7 (0.8)	446	23.4 (0.4)	4297	
Heavy alcohol drinking	6.9 (0.6)	205	13.9 (0.3)	2702	<.0001
Hypertension	41.3 (1.0)	1695	24.0 (0.4)	6109	<.0001
Diabetes	18.5 (0.8)	725	8.6 (0.2)	2199	<.0001
Physical activity					
<600 MET-minutes/wk	64.7 (1.1)	2257	48.6 (0.5)	10,847	<.0001
≥600 MET-minutes/wk	35.3 (1.1)	1063	51.4 (0.5)	10,137	
Right HGS (kg), mean (SE)	18.5 (0.1)	-	32.6 (0.1)	-	<.0001
Left HGS (kg), mean (SE)	18.8 (0.2)	-	31.0 (0.1)	-	<.0001
Dominant HGS (kg), mean (SE)	18.4 (0.1)	-	32.7 (0.1)	-	<.0001
BUN (mg/dL), mean (SE)	15.7 (0.1)	-	14.1 (0.0)	-	<.0001
Creatinine (mg/dL), mean (SE)	0.8 (0.0)	-	0.8 (0.0)	-	.8060
eGFR (mL/min/1.73 m^2^), mean (SE)	90.2 (0.5)	-	99.1 (0.2)	-	<.0001
CKD Stages 1-2	91.4 (0.5)	3215	98.3 (0.1)	21,584	<.0001
CKD Stages 3a-5	8.6 (0.5)	388	1.7 (0.1)	559	
Proteinuria	3.0 (0.3)	111	1.7 (0.1)	377	<.0001

CKD = chronic kidney disease, BMI = body mass index, BUN = blood urea nitrogen, eGFR = estimated glomerular filtration rate, HGS = hand grip strength, LMS = low muscle strength, MET = metabolic equivalents, NMS = normal muscle strength, SD = standard deviation.

In men, LMS defined as HGS < 28.9 kg, and NMS as HGS ≥ 28.9 kg; in women, LMS defined as HGS < 16.8 kg, and NMS as HGS ≥ 16.8 kg.

CKD Stages 1-2 defined as eGFR ≥ 60 mL/min/1.73 m^2^, and CKD Stages 3a-5 as eGFR < 60 mL/min/1.73 m^2^.

**Table 3:** Characteristics of the study population according to gender.

Characteristic	Men					Women				
	LMS(N = 1546)		NMS(N = 10,043)		*P* value	LMS(N = 2057)		NMS(N = 12,100)		*P* value
	Weighted% (SE)	UnweightedN	Weighted% (SE)	UnweightedN		Weighted% (SE)	UnweightedN	Weighted% (SE)	UnweightedN	
Age (yrs), mean (SE)	58.1 (0.7)	-	44.1 (0.2)	-	<.0001	59.0 (0.6)	-	45.3 (0.2)	-	<.0001
<65	51.3 (1.6)	520	90.7 (0.3)	8237	<.0001	51.3 (1.5)	862	88.1 (0.3)	9986	<.0001
≥65	48.7 (1.6)	1026	9.3 (0.3)	1806		48.7 (1.5)	1195	11.9 (0.3)	2114	
Household income										
Low	33.3 (1.6)	487	23.5 (0.6)	2261	<.0001	30.7 (1.3)	615	24.3 (0.6)	2796	<.0001
Lower middle	24.0 (1.4)	381	25.6 (0.6)	2523		24.4 (1.1)	506	25.1 (0.5)	3031	
Higher middle	22.0 (1.2)	343	25.5 (0.6)	2597		22.1 (1.1)	449	25.3 (0.5)	3107	
High	20.8 (1.5)	319	25.4 (0.7)	2633		22.8 (1.2)	475	25.4 (0.7)	3120	
Educational level										
≤Elementary school	29.5 (1.5)	553	7.3 (0.3)	1000	<.0001	47.6 (1.7)	1054	14.5 (0.4)	2226	<.0001
Middle school	13.5 (1.0)	212	7.7 (0.3)	919		9.5 (0.8)	187	8.9 (0.3)	1155	
High school	33.4 (1.6)	392	39.0 (0.7)	3429		22.1 (1.3)	335	36.6 (0.6)	3925	
≥College	23.6 (1.5)	278	46.0 (0.8)	4080		20.8 (1.3)	331	40.1 (0.7)	4261	
Weight (kg), mean (SE)	65.2 (0.4)	-	72.9 (0.1)	-	<.0001	55.3 (0.2)	-	58.5 (0.1)	-	<.0001
Height (cm), mean (SE)	166.3 (0.2)	-	172.1 (0.1)	-	<.0001	153.3 (0.2)	-	158.8 (0.1)	-	<.0001
BMI (kg/m^2^), mean (SE)	23.5 (0.1)	-	24.6 (0.0)	-	<.0001	23.6 (0.1)	-	23.2 (0.0)	-	.0010
<25	69.9 (1.5)	1112	58.3 (0.6)	5827	<.0001	68.2 (1.2)	1349	73.2 (0.5)	8601	<.0001
≥25	30.1 (1.5)	426	41.7 (0.6)	4207		31.8 (1.2)	694	26.8 (0.5)	3482	
Smoking										
Never	28.0 (1.5)	369	25.4 (0.5)	2302	<.0001	90.5 (0.9)	1829	87.8 (0.4)	10,577	.0220
Past	44.5 (1.5)	760	35.1 (0.6)	3871		4.7 (0.6)	82	6.3 (0.3)	704	
Current	27.4 (1.4)	368	39.5 (0.6)	3664		4.8 (0.6)	78	6.0 (0.3)	633	
Heavy alcohol drinking	10.9 (1.0)	147	21.1 (0.5)	2032	<.0001	3.9 (0.6)	58	6.2 (0.3)	670	.0040
Hypertension	40.3 (1.5)	733	28.0 (0.5)	3210	<.0001	42.0 (1.4)	962	19.7 (0.5)	2899	<.0001
Diabetes	19.9 (1.2)	345	10.1 (0.3)	1217	<.0001	17.4 (1.0)	380	7.1 (0.3)	982	<.0001
Physical activity										
<600 MET-min/wk	60.0 (1.7)	914	45.5 (0.7)	4537	<.0001	68.3 (1.3)	1343	52.0 (0.6)	6310	<.0001
≥600 MET-min/wk	40.0 (1.7)	511	54.5 (0.7)	4889		31.7 (1.3)	552	48.0 (0.6)	5248	
Right HGS (kg), mean (SE)	24.5 (0.1)	-	40.5 (0.1)	-	<.0001	13.8 (0.1)	-	24.0 (0.1)	-	<.0001
Left HGS (kg), mean (SE)	25.1 (0.2)	-	38.7 (0.1)	-	<.0001	13.9 (0.1)	-	22.6 (0.1)	-	<.0001
Dominant HGS (kg), mean (SE)	24.4 (0.1)	-	40.6 (0.1)	-	<.0001	13.8 (0.1)	-	24.0 (0.1)	-	<.0001
BUN (mg/dL), mean (SE)	16.5 (0.3)	-	14.7 (0.0)	-	<.0001	15.1 (0.1)	-	13.3 (0.0)	-	<.0001
Creatinine (mg/dL), mean (SE)	1.0 (0.0)	-	1.0 (0.0)	-	.1680	0.7 (0.0)	-	0.7 (0.0)	-	.0020
eGFR (mL/min/1.73 m^2^), mean (SE)	88.0 (0.7)	-	96.6 (0.2)	-	<.0001	91.9 (0.6)	-	101.8 (0.2)	-	<.0001
CKD stages 1-2	90.0 (0.8)	1337	98.2 (0.1)	9733	<.0001	92.6 (0.7)	1878	98.5 (0.1)	11,851	<.0001
CKD stages 3a-5	10.0 (0.8)	209	1.8 (0.1)	310		7.4 (0.7)	179	1.5 (0.1)	249	
Proteinuria	4.3 (0.6)	72	2.0 (0.2)	228	<.0001	2.0 (0.4)	39	1.4 (0.1)	149	.0700

CKD = chronic kidney disease, BMI = body mass index, BUN = blood urea nitrogen, eGFR = estimated glomerular filtration rate, HGS = hand grip strength, LMS = low muscle strength, MET = metabolic equivalents, SD = standard deviation.

In men, LMS defined as HGS < 28.9 kg, and NMS as HGS ≥ 28.9 kg; in women, LMS defined as HGS < 16.8 kg, and NMS as HGS ≥ 16.8 kg.

CKD Stages 1-2 defined as eGFR ≥ 60 mL/min/1.73 m^2^, and CKD Stages 3a-5 as eGFR < 60 mL/min/1.73 m^2^.

To determine the association between renal function and the prevalence of LMS in all subjects, logistic regression analysis was performed, and the results are presented in Table 4. The subjects with eGFR < 60 mL/min/1.73 m^2^ were at a higher risk of developing LMS than those with eGFR ≥ 60 mL/min/1.73 m^2^ after adjusting for age (OR, 1.772; 95% CI, 1.498–2.096), and the significant differences still existed after further adjusting for other factors including age. Similar tendencies were shown in men and women when analyzed according to gender; however, the risk of developing LMS was higher in men than in women.

**Table 4:** Odds ratios for muscle strength in total subjects.

Subgroups	Model 1 [OR (95% CI)]	Model 2 [OR (95% CI)]	Model 3 [OR (95% CI)]	Model 4 [OR (95% CI)]	Model 5 [OR (95% CI)]
Total					
CKD stages 1-2	Reference	Reference	Reference	Reference	Reference
CKD stages 3a-5	5.577 (4.749-6.550)	1.772 (1.498-2.096)	1.822 (1.543-2.152)	1.865 (1.580-2.201)	2.043 (1.661-2.514)
Men*					
CKD stages 1-2	Reference	Reference	NA	Reference	Reference
CKD stages 3a-5	5.969 (4.792-7.436)	1.892 (1.503-2.382)	NA	1.970 (1.561-2.486)	2.535 (1.895-3.391)
Women*					
CKD stages 1-2	Reference	Reference	NA	Reference	Reference
CKD stages 3a-5	5.435 (4.297-6.875)	1.727 (1.350-2.208)	NA	1.770 (1.385-2.262)	1.563 (1.168-2.091)

CKD = chronic kidney disease, NA = not available, OR = odds ratio.

Odds ratios with adjustments using logistic regression models.

* Not adjusted for gender.

Model 1: unadjusted.

Model 2: adjusted for age.

Model 3: adjusted for age and gender.

Model 4: adjusted for age, gender, and BMI.

Model 5: adjusted for age, gender, household income, educational level, BMI, smoking, heavy alcohol drinking, hypertension, diabetes, physical activity, BUN, and proteinuria.

The results from the logistic regression analysis in all subjects according to BMI are presented in Table 5. The subjects with eGFR < 60 mL/min/1.73 m^2^ and BMI < 25 kg/m^2^ were at a higher risk for LMS after adjusting for age (OR, 1.956; 95% CI, 1.583–2.416) than those with eGFR ≥ 60 mL/min/1.73 m^2^ and BMI < 25 kg/m^2^. A similar tendency was shown in the subjects with BMI ≥ 25 kg/m^2^; however, the OR of LMS for them was reduced (OR, 1.586; 95% CI, 1.210–2.079). In men, the ORs of LMS were higher in the subjects with eGFR < 60 mL/min/1.73 m^2^ than in those with eGFR ≥ 60 mL/min/1.73 m^2^ when adjusted for age, regardless of BMI. In women, the ORs of LMS were also higher in the subjects with eGFR < 60 mL/min/1.73 m^2^ than in those with eGFR ≥ 60 mL/min/1.73 m^2^ when adjusted for age, regardless of BMI; however, the OR of the subjects with BMI < 25 kg/m^2^ was higher than that of their counterparts.

**Table 5:** Odds ratios for muscle strength in total subjects according to BMI and gender.

Subgroups	Model 1 [OR (95% CI)]	Model 2 [OR (95% CI)]	Model 3 [OR (95% CI)]	Model 4 [OR (95% CI)]
Total				
<25 kg/m^2^				
CKD stages 1-2	Reference	Reference	Reference	Reference
CKD stages 3a-5	6.304 (5.111-7.776)	1.956 (1.583-2.416)	1.990 (1.609-2.460)	1.942 (1.491-2.529)
≥25 kg/m^2^				
CKD stages 1-2	Reference	Reference	Reference	Reference
CKD stages 3a-5	4.866 (3.746-6.321)	1.586 (1.210-2.079)	1.667 (1.266-2.195)	2.268 (1.632-3.154)
Men*				
<25 kg/m^2^				
CKD stages 1-2	Reference	Reference	NA	Reference
CKD stages 3a-5	6.896 (5.220-9.110)	2.008 (1.494-2.700)	NA	2.505 (1.722-3.643)
≥25 kg/m^2^				
CKD stages 1-2	Reference	Reference	NA	Reference
CKD stages 3a-5	4.630 (3.200-6.699)	1.913 (1.315-2.784)	NA	2.990 (1.839-4.863)
Women*				
<25 kg/m^2^				
CKD stages 1-2	Reference	Reference	NA	Reference
CKD stages 3a-5	5.800 (4.250-7.914)	1.865 (1.370-2.539)	NA	1.389 (0.963-2.002)
≥25 kg/m^2^				
CKD stages 1-2	Reference	Reference	NA	Reference
CKD stages 3a-5	4.704 (3.295-6.715)	1.501 (1.023-2.202)	NA	1.864 (1.191-2.917)

CKD = chronic kidney disease, NA = not available, OR = odds ratio.

Odds ratios with adjustments using logistic regression models.

* Not adjusted for gender.

Model 1: unadjusted.

Model 2: adjusted for age.

Model 3: adjusted for age and gender.

Model 4: adjusted for age, gender, household income, educational level, smoking, heavy alcohol drinking, hypertension, diabetes, physical activity, BUN, and proteinuria.

The results from the logistic regression analysis in all subjects by age are summarized in Table 6. The subjects with eGFR < 60 mL/min/1.73 m^2^ and aged < 65 years had a higher risk for LMS after controlling for gender and BMI (OR, 2.193; 95% CI, 1.242–3.875) than those with eGFR ≥ 60 mL/min/1.73 m^2^ and aged < 65 years. This tendency remained in the subjects aged ≥ 65 years, but the OR of LMS for them was reduced (OR, 1.684; 95% CI, 1.408–2.013). In men, the ORs of LMS were higher in the subjects with eGFR < 60 mL/min/1.73 m^2^ than in those with eGFR ≥ 60 mL/min when adjusted for BMI, regardless of age. However, the OR of the subjects aged < 65 years was higher than that of their counterparts. In women aged < 65 years, there was no statistically significant difference, whereas the OR of LMS in their counterparts was higher in the subjects with eGFR < 60 mL/min/1.73 m^2^ than in those with eGFR ≥ 60 mL/min/1.73 m^2^ when controlling for BMI.

**Table 6:** Odds ratios for muscle strength in total subjects according to age and gender.

Subgroups	Model 1 [OR (95% CI)]	Model 2 [OR (95% CI)]	Model 3 [OR (95% CI)]	Model 4 [OR (95% CI)]
Total				
<65 yrs				
CKD stages 1-2	Reference	Reference	Reference	Reference
CKD stages 3a-5	2.016 (1.148-3.542)	2.153 (1.220-3.799)	2.193 (1.242-3.875)	1.232 (0.632-2.401)
≥65 yrs				
CKD stages 1-2	Reference	Reference	Reference	Reference
CKD stages 3a-5	1.594 (1.335-1.903)	1.608 (1.347-1.919)	1.684 (1.408-2.013)	1.376 (1.113-1.700)
Men*				
<65 years				
CKD stages 1-2	Reference	NA	Reference	Reference
CKD stages 3a-5	2.658 (1.351-5.232)	NA	2.711 (1.375-5.344)	1.713 (0.764-3.839)
≥65 yrs				
CKD stages 1-2	Reference	NA	Reference	Reference
CKD stages 3a-5	1.567 (1.225-2.004)	NA	1.676 (1.309-2.146)	1.456 (1.090-1.943)
Women*				
<65 yrs				
CKD stages 1-2	Reference	NA	Reference	Reference
CKD stages 3a-5	1.379 (0.484-3.924)	NA	1.392 (0.488-3.972)	0.676 (0.185-2.472)
≥65 yrs				
CKD stages 1-2	Reference	NA	Reference	Reference
CKD stages 3a-5	1.649 (1.275-2.133)	NA	1.708 (1.320-2.210)	1.261 (0.930-1.712)

CKD = chronic kidney disease, NA = not available, OR = odds ratio.

Odds ratios with adjustments using logistic regression models.

* Not adjusted for gender.

Model 1: unadjusted.

Model 2: adjusted for gender.

Model 3: adjusted for gender and BMI.

Model 4: adjusted for gender, household income, educational level, BMI, smoking, heavy alcohol drinking, hypertension, diabetes, physical activity, BUN, and proteinuria.

## 4. Discussion

This study investigated the relationships between renal function and HGS, a surrogate marker of muscular strength, among adults using representative and reliable data for the Korean population. The principle findings were that in general, LMS was more prevalent in the subjects with eGFR < 60 mL/min/1.73 m^2^ than in those with eGFR ≥ 60 mL/min/1.73 m^2^, and the prevalence rate of LMS appeared to be generally higher in men than in women. This gender difference may be partially explained by a stricter definition of LMS in men than in women. To the best of our knowledge, studies on the associations between renal function and HGS have been rarely conducted in Korean adults. It may be meaningful that the results from this study could be used to provide better knowledge for the development of healthcare strategies that support the management of patients with decreased renal function based on HGS in clinical or public health practice.

The overall prevalence rate of LMS was 1.772 times higher in the subjects with eGFR < 60 mL/min/1.73 m^2^ than in their counterparts after adjusting for age, and the pattern was observed after adjusting for additional variables. Similar patterns were also shown in the subgroup analyses according to gender. This is consistent with the findings of previous studies.^[4–6]^ Hiraki et al reported that HGS was significantly lower in patient with CKD stages 4 and 5 than in those with CKD stages 2 and 3.^[6]^ The average HGS was reduced from 35.2 kg among patients with CKD stage 2 to 22.4 kg among those with CKD stage 5.^[6]^ According to the study conducted by Moon et al with 11,625 Koreans aged ≥ 40 years using the KNHANES 2008–2011, the prevalence of sarcopenia characterized by progressive loss of muscle mass and strength was higher in those with even mildly diminished kidney functions.^[4]^ The prevalence of sarcopenia was 4.3% in normal and CKD stage 1, 6.3% in CKD stage 2, and 15.4% in CKD stages 3–5.^[4]^ These results can be explained by several characteristics (e.g., anemia, decrease in serum albumin and hemoglobin levels, presence of proteinuria, protein hypercatabolism, advanced age, and inflammation) that usually appear in CKD patients.^[21]^ Ultimately, these characteristics lead to the reduction in muscle mass and strength.^[21]^ In this study, the subjects with LMS were older than those with NMS, and the prevalence rate of proteinuria was higher in the subjects with LMS than in their counterparts.

The subjects with BMI < 25 kg/m^2^ and eGFR < 60 mL/min/1.73 m^2^ had a higher risk for LMS than those with BMI ≥ 25 kg/m^2^ and eGFR < 60 mL/min/1.73 m^2^ after controlling for age, and this tendency also remained in the subgroup analyses stratified by gender. This result may conflict with the effects of BMI on renal function and HGS. According to a previous study in England,^[13]^ higher BMI was associated with an increased risk for advanced CKD, regardless of the presence of HTN, DM, or cardiovascular diseases, which can substantially affect renal function. Subsequently, reduced renal function was likely to have an increased negative effect on HGS as shown in the previous studies.^[4–6]^ In contrast, Pasdar et al reported that HGS increased significantly with increasing BMI in both men and women, but the association of HGS with BMI was stronger in men than in women.^[22]^ Additionally, this association was more significant in the obese group (BMI ≥ 30 kg/m^2^) than in the overweight group (25 kg/m^2^ ≤ BMI < 30 kg/m^2^).^[22]^ Taken together, BMI can negatively affect HGS directly or indirectly through reduction in renal function. However, this pattern was changed after adjusting for additional variables. LMS was more prevalent in subjects with BMI ≥ 25 kg/m^2^ and eGFR < 60 mL/min/1.73 m^2^ than in those with BMI < 25 kg/m^2^ and eGFR < 60 mL/min/1.73 m^2^.

Interestingly, LMS was more prevalent in men aged < 65 years with eGFR < 60 mL/min/1.73 m^2^ than in those aged ≥ 65 years with eGFR < 60 mL/min/1.73 m^2^ after adjusting for BMI. No statistically significant difference was found in women aged < 65 years with eGFR < 60 mL/min/1.73 m^2^ after adjusting BMI; however, LMS was more prevalent in those aged ≥ 65 years with eGFR < 60 mL/min/1.73 m^2^. These results could be explained to some extent by the prevalence rates of HTN and DM, which can negatively affect renal function, based on gender. According to a study conducted with the data of the 5^th^ and 6^th^ KNHANES, Choi et al reported that women tended to have more HTN than men after 60 years of age.^[23]^ Li et al also presented that the prevalence rate of DM was higher among men aged 45–54 years, whereas DM was more prevalent among women aged 65–74 years.^[24]^ These tendencies in HTN and DM status were likely to contribute to decreased renal function at difference periods depending on gender, thereby leading to lower HGS at different times. However, after adjusting for additional variables, including HTN and DM, this phenomenon was rarely observed.

Age and gender, non-modifiable factors, were found to be important determinants of HGS in the previous studies.^[25–32]^ HGS tended to decrease with increasing age in both men and women. In the present study, the peak HGS was shown between 30–40 years of age in both genders; it remained or was slightly reduced between 40–50 years of age, and then its decline began to accelerate after age 50 years (see Figure, Supplemental Digital Content 1, http://links.lww.com/MD/H555, which illustrates life course profiles of HGS for Korean women and men). However, the decline was more rapid among men than women in late life. This result is consistent with those from the previous studies.^[25,32]^ HGS in the present study was also much higher in men than women as shown in the previous studies.^[25,27,29,32]^ Besides age and gender, chronic conditions (e.g., HTN, DM, and dyslipidemia), life styles, and nutritional status are likely to worsen loss of muscle strength and mass directly or indirectly via their negative effects on renal function. For example, according to a previous study conducted using the data from the KNHANES (2014–2015),^[33]^ Yi et al demonstrated that there was a highly significant inverse association between relative HGS (defined as the absolute HGS divided by BMI) and the risk of metabolic syndrome. In addition, Lee et al conducted the study using the same data and reported that HGS (normalized to body weight) was inversely associated with the prevalence rate of type 2 DM as well as the fasting glucose level and HbA1c level.^[34]^ A similar result was reported in the study using the data from the National Health and Nutrition Examination Survey (NHANES) of the USA.^[35]^ Consequently, considering these various factors, which may potentially affect the association between HGS and kidney function, helps to develop better strategies to utilize such associations when estimating renal function in clinical or public health practice.

The present study has some limitations, which should be considered when the results are interpreted. First, it is difficult to clearly conclude the causality between renal function and HGS due to the drawback of the cross-sectional design. Second, the overall prevalence rates of decreased renal function and LMS were most likely underestimated, since the subjects with incomplete information on HGS measurements and serum creatinine were excluded. However, this process was unlikely to have an effect on the study results because it is highly possible that the missingness randomly occurred. Third, almost all of variables used to evaluate the effects of renal function on HGS were measured at a single time point, which would negatively affect data accuracy. Fourth, the sociodemographic characteristics of the study subjects were collected using the survey, which might reflect recall bias to some extent. Finally, various formulae for calculating eGFR (e.g., Cockcroft-Gault and Modification of Diet in Renal Disease) are currently available, so using other formulae instead of CKD epidemiology collaboration may lead to slightly different results than those revealed in this study.

## 5. Conclusion

The results showed that decreased renal function was likely to contribute to an increased prevalence rate of LMS in terms of HGS. Age, gender, and BMI also had significant effects on the association between reduced renal function and LMS. This association is likely to assist in developing better strategies to estimate renal function in clinical or public health practice.

## Acknowledgments

This study was supported by a research fund from Chosun University, 2019. Chosun University had no role in the design, methods, data collection, analysis and preparation of paper.

## Author contributions

Young-Mo Yang conceptualized and designed the study, obtained and analyzed the data, interpreted the results, and drafted the original article. Eun Joo Choi conceptualized and designed the study, interpreted the results, and critically revised the draft with Dr Yang. Both authors have read and approved of the final version.

**Conceptualization:** Young-Mo Yang, Eun Joo Choi.

**Data curation:** Young-Mo Yang.

**Formal analysis:** Young-Mo Yang.

**Funding acquisition:** Eun Joo Choi.

**Investigation:** Young-Mo Yang, Eun Joo Choi.

**Methodology:** Young-Mo Yang.

**Project administration:** Eun Joo Choi.

**Resources:** Young-Mo Yang, Eun Joo Choi.

**Software:** Young-Mo Yang, Eun Joo Choi.

**Validation:** Young-Mo Yang, Eun Joo Choi.

**Visualization:** Young-Mo Yang, Eun Joo Choi.

**Writing – original draft:** Young-Mo Yang, Eun Joo Choi.

**Writing – review & editing:** Young-Mo Yang.

## Supplementary Material


