# Supplementary material for: Association of renal function with muscle strength in Korean adults: A population-based study using the Korea National Health and Nutrition Examination Surveys (KNHANES) from 2014 to 2018
Source: Medicine (Baltimore). 2022 Oct 14;101(41):e31014. doi: 10.1097/MD.0000000000031014 (PMC9575765; doi:10.1097/MD.0000000000031014)

Supplemental Digital Content 1. Figure that illustrates life course profiles of hand grip strength (HGS) for Korean women and men. Tif

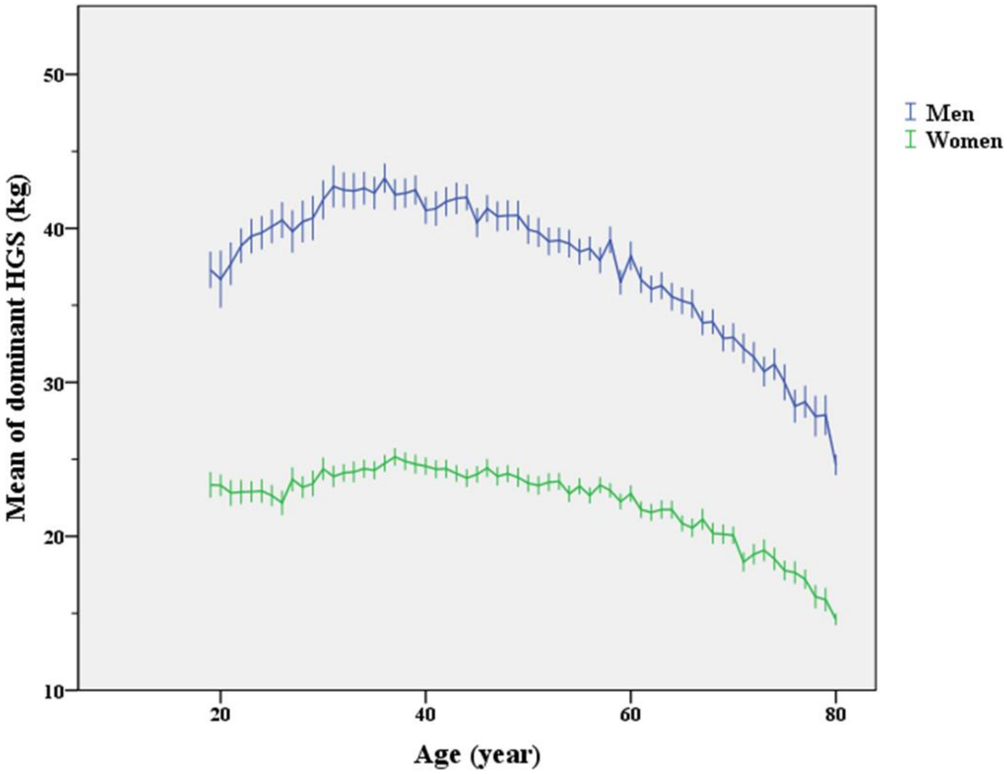

Supplement: Supplementary file 1 [file medi-101-e31014-s001.pdf]
